# Put rights at the centre of person‐ and people‐centred HIV prevention

**DOI:** 10.1002/jia2.70028

**Published:** 2025-10-08

**Authors:** Megan McLemore, Joseph J. Amon

**Affiliations:** ^1^ Independent Consultant Jacksonville Florida USA; ^2^ Department of Epidemiology Center for Public Health and Human Rights Johns Hopkins Bloomberg School of Public Health Baltimore Maryland USA

**Keywords:** HIV, human rights, people‐centred care, Jamaica, Indonesia, Mozambique

## Abstract

**Introduction:**

“Person‐centred” and “people‐centred” HIV prevention programmes both seek to scale up access to HIV prevention services. A “person‐centred” approach presents a vision of a client with agency in decision‐making, engaged and empowered, working with providers in a process that is not disease‐centric but focused on addressing, holistically, a client's needs. A “people‐centred” approach recognizes the broader role of family and community, as well as the influence of the political and legal environment as barriers or facilitators to HIV services. In both cases, human rights are a critical determinant of positive or negative outcomes.

**Discussion:**

In 2017, the Global Fund's *Breaking Down Barriers* initiative funded baseline assessments in 20 countries examining key human rights barriers to HIV services. Subsequent evaluations in 2019–2021 and 2022–2024 focused on the scale‐up of community‐led human rights interventions and the impact of these programmes on access to HIV prevention and care. Results from the latest assessment describe a range of strategies and impact across diverse countries, settings and populations. For example, in Indonesia, transgender‐led organizations catalysed a national drive to allow transgender persons to receive gender‐matched identity cards, allowing thousands of individuals to access HIV prevention and treatment and broader social benefits. In Mozambique, peer‐led paralegals and community advocates promoted legal literacy and assisted clients with claims of human rights violations, preventing access to HIV services. In Jamaica, lesbian, gay, bisexual and transgender led organizations sponsored trainings that advanced community activism for HIV prevention, education and advocacy. Despite facing stigma and challenging legal environments, in each case, human rights‐based programmes removed structural and legal barriers to HIV prevention services, strengthening accountability and increasing uptake and retention in HIV services, especially among marginalized and criminalized populations.

**Conclusions:**

Community mobilization led by key populations is a long‐term undertaking that requires partnership and support from a wide range of stakeholders to ensure sustainability. A growing body of evidence across a range of diverse countries and settings demonstrates the impact of rights‐based and people‐centred programmes on access to, and retention in, HIV prevention and treatment.

## 1 INTRODUCTION

The past decade has seen the global HIV response move towards a model of “person‐centred” services for prevention and treatment [1]. At the heart of this approach is a vision of a client with agency in decision‐making, engaged and empowered, working with providers in a process that is not disease‐centric but focused on holistically addressing a client's needs [2]. Increasingly, however, there is recognition that a broader “people‐centred” approach, that emphasizes the role of family, community, environment and other factors, such as the human rights environment in which HIV services are delivered, is critical to ensuring access to HIV prevention and treatment [3, 4, 5].

For many decades, the HIV response has pioneered aspects of this broader people‐centred approach – engaging communities and underlining the importance of addressing and overcoming barriers that limit access to prevention and care, including human rights abuses which disproportionately affect adolescent girls and young women, sex workers, lesbian, gay, bisexual, transgender, queer and intersex (LGBTQI) individuals and people who inject drugs (PWID). Collectively, these key populations (KPs) account for at least 70% of new HIV acquisition worldwide and human rights abuses represent a major driver of HIV risk, as a result of discrimination in healthcare and community settings; the criminalization of these populations; and the experience of violence—from police, clients and community members [6].

To address this, there needs to be at the core of people‐centred approaches evidence‐based human rights interventions operating at scale and working at multiple levels: addressing individual rights violations, community‐level stigma and discrimination, and punitive laws that target KPs and drive individuals away from prevention and treatment. These laws, as currently proposed and implemented, for example, in Uganda, Ghana and Kenya, can also target HIV organizations by outlawing the “promotion” or “normalization” of homosexuality [7]. In other countries, prevention approaches such as the distribution and possession of condoms or sterile syringes can be used as evidence of crimes such as solicitation, prostitution or illicit drug use [8, 9].

Since 2017, the Global Fund's *Breaking Down Barriers* (BDB) initiative has supported community‐led programming in more than 20 countries seeking to reduce human rights‐related barriers to HIV, tuberculosis (TB) and malaria prevention and treatment [10]. These programmes seek to mobilize communities and ensure accountability and access to justice in the face of rights violations. More specifically, programmes aim to eliminate stigma and discrimination; ensure rights‐based law enforcement practices; promote legal literacy; increase access to justice; and improve laws, regulations and polices relating to HIV (see Box).

**Box. Breaking Down Barriers initiative: programmes to remove human rights‐related barriers to HIV services**

- Eliminating stigma and discrimination in all settings
- Ensuring non‐discriminatory provision of healthcare
- Ensuring rights‐based law enforcement practices
- Legal literacy (“know your rights”)
- Increasing access to justice
- Improving laws, regulations and polices relating to HIV
- Reducing gender discrimination, harmful gender norms and violence against women and girls in all their diversity
- Community mobilization and advocacy for human rights

By design, none of these interventions can be undertaken without engaging multiple stakeholders, including national health ministries and other government actors, civil society and non‐governmental organizations (NGOs), the Joint United Nations Programme on HIV/AIDS (UNAIDS), international donors and technical partners. Community and peer leadership is prioritized in programme design and implementation, reflecting the Global Fund definition of “people‐centred” care that puts “people *and communities* (emphasis added) at the centre of services” [11].

Without addressing structural and legal barriers to HIV services, violence, discrimination, lack of access to prevention and denial of care and treatment will continue to impact marginalized and criminalized populations [12]. Human rights‐based interventions which seek to create an enabling environment for HIV programmes can expand access to both biological (e.g. pre‐exposure prophylaxis or PrEP) and behavioural HIV prevention strategies and reduce loss to follow‐up and ensure ongoing use of HIV prevention methods.

To illustrate the importance of integrating human rights‐based interventions into people‐centred HIV programmes, we highlight results from three country assessments conducted in Indonesia, Mozambique and Jamaica, and describe in each case: (1) how community‐based organizations led rights‐based campaigns to address issues that they identified as posing critical barriers to HIV prevention; (2) the challenges they faced and the strategies used to overcome those challenges; and (3) the direct and indirect outcomes resulting from the campaigns.

## 2 DISCUSSION

Between 2022 and 2024, an evaluation of the *Breaking Down Barriers* initiative was conducted to examine community‐led, and people‐centred, human rights‐based HIV programmes in 20 countries [13]. The evaluation used an implementation learning approach including a document review of programme monitoring and budget documents (such as grant agreements and related documents); financial and programmatic reports; programme outputs (activity reports, tools, training manuals, guidelines, policies, etc.); documentation on human rights‐related barriers (violations reports, press statements, etc.); and other documents related to national strategies and programmes to reduce or remove human rights barriers.

Key informant interviews were conducted with implementers, government officials, human rights experts and beneficiaries, and sought to understand challenges faced in the implementation and scale‐up of rights‐based initiatives, as well as key outputs, outcomes and impact. Interviewers probed strategies to advance HIV programmes in the face of challenging legal and political environments and to assess different perspectives on priorities for future investment. In all countries, stakeholder validation meetings were held to share preliminary results and receive feedback.

### 2.1 Indonesia

In Indonesia, the Breaking Down Barriers assessment was conducted between December 2022 and February 2023 and included interviews with 190 individuals, including programme implementers, representatives from government agencies, and community and technical partners. Site visits were conducted in Bandung, Bogor, Jakarta (Java) and Medan (Sumatra). Follow‐up interviews were conducted in March 2023, and stakeholder validation meetings were held in April 2023 [14].

One programme the evaluation highlighted was the collaborative work of the transgender‐led network “Our Voice,” the Indonesia AIDS Coalition (a community‐based organization focused on promoting the rights of people living with HIV and KPs), and the Indonesian government to address the challenges KPs face if they did not have a national identity card (Kartu Tanda Penduduk or KTP).

The KTP card gives Indonesians access to HIV prevention and treatment services, as well as critical health and social welfare programmes [15, 16, 17, 18]. Having a KTP card ensures affordable access to HIV testing, PrEP and syringe access programmes, yet transgender individuals face specific obstacles to obtaining the ID cards, as many leave their families and hometowns before obtaining a card, or the card they hold no longer matches their gender identity, resulting in stigma and denial of access to HIV services.

Initially, HIV and LGBT organizations worked with local government officials to facilitate access to the KTP for individuals. However, all groups recognized that a more durable solution was needed, and together they conducted advocacy with the Ministry of Home Affairs, which issued new guidance that loosened restrictions on eligibility for national ID cards for transgender people. This guidance then became the basis of a letter to civil registration agencies around the country.

What was needed next was community mobilization to take advantage of the changing requirements. Supported by multiple donors, a coalition led by Our Voice obtained 897 national identity cards for transgender people in 29 districts in the first year following the regulatory change [14]. Key participants in the coalition were advocacy officers and paralegals from “District Task Forces” established as part of the BDB initiative in 23 high‐burden districts for HIV. In some cases, District Task Forces also included community leaders, government officials and other local stakeholders. Advocacy Officers, who were members of the KPs they served, worked with peer advocates to inform the community of the new eligibility rules, to collect and develop necessary documentation, and help individuals submit the documents to the appropriate civil agencies.

District Task Force teams also successfully campaigned for increased access to ID cards for female sex workers. A 2021 study indicated that having a KTP was a key factor in the ability of female sex workers in Indonesia to access HIV and viral load testing and that having a KTP correlated with adherence to treatment regimens [18]. As of December 2022, at least 100 KP members had enrolled in the health insurance programme as a result of obtaining national ID cards, and community advocacy on this issue was ongoing [14].

Of course, challenges and barriers to HIV prevention for KPs in Indonesia remain. The evaluation found widespread concern among implementers about the impact of a newly proposed Criminal Code on the rights of women, LGBT persons, and on HIV prevention and treatment services [14]. Among other things, the law criminalizes consensual extramarital sex and distributing information about contraception to minors [19]. As same‐sex couples cannot marry in the country, all same‐sex conduct would be against the law. People who use drugs also face severe treatment, especially women, by Indonesia's drug laws. One study found that among women in Indonesia who have come into contact with the police because of injection drug use, 90% also have experienced extortion, physical and/or sexual violence [20].

### 2.2 Mozambique

In Mozambique, the BDB assessment was conducted between January and April 2023 and included 26 key informant interviews and group discussions with representatives of KPs, including sex workers, people who use or inject drugs (PWUD/PWID), gay men and other men who have sex with men (MSM) and transgender people. Preliminary results and stakeholder validation meetings were held in November 2023 [21].

A key component of the Mozambique programme was the development of an integrated structure of support for people experiencing stigma and discrimination in access to HIV programmes and health services. The initiative was championed by the Fundação para o Desenvolvimento da Comunidade (Foundation for Community Development, or FDC), a non‐profit organization that promotes social justice and works to empower communities to overcome poverty.

To address these challenges, FDC, the Centro de Colaboracao em Saude (CSS) and other NGOs worked with local government officials, police and community health committees, to resolve barriers to HIV services through the training and engagement of KP members as *activistas* and paralegals who could conduct legal literacy trainings and educate KPs and affected communities in their rights to HIV prevention. When necessary, *activistas* and paralegals also worked closely with district supervisors or provincial human rights officials to escalate concerns, such as denial of care and discriminatory or abusive treatment, to the Mozambique government's legal aid institute or directly to prosecutors [21].

Although time‐consuming and requiring sustained engagement, the organizations participating reported positive results in reconnecting people to HIV prevention and treatment programmes. For example, the Centro de Colaboracao em Saude (CSS), a leading implementer alongside FDC, reported that from January to March 2020, 696 of 875 patients (79%) were reintegrated into HIV care by health advocates, including paralegals who provided assistance in cases involving violations of human rights. Between July and October of 2021, 591 people living with HIV returned to treatment following interventions from *activistas* and paralegals [22].

**Figure 1:**
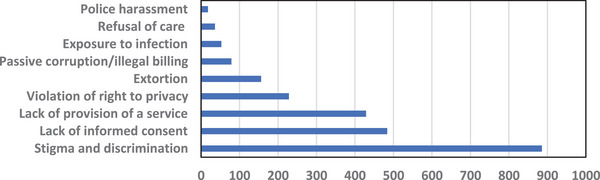
Human rights claims related to access to HIV services reported to Fundação para o Desenvolvimento da Comunidade, Mozambique (July 2022–January 2023).

FDC reported a total of 7381 human rights‐related claims were handled by its team of 10 lawyers, 300 paralegals and 15,000 *activistas* between July 2022 and January 2023. Nearly a third of all claims (2369) were related to human rights violations experienced in health facilities. Stigma and discrimination represented the single largest number of complaints, followed by lack of informed consent and lack of access to care. Other complaints included violations of the right to privacy, extortion, corruption and police harassment, which impact both HIV prevention and care (see Figure 1) [23].

According to reports by the U.S. President's Emergency Plan for AIDS Relief (PEPFAR), assistance by *activistas* and paralegal advocates encouraged clients to seek redress for inappropriate or discriminatory treatment and increased accountability from both clinic staff and the health system more generally [24].

### 2.3 Jamaica

In Jamaica, the BDB assessment was conducted between August and November 2023, with interviews conducted remotely and during a country visit in October 2023. The research team interviewed 24 key implementers, government agency representatives, technical partners and beneficiaries. Stakeholders were given preliminary findings and recommendations for review and comment in October 2023, prior to finalization of the report [25].

Since the start of the HIV epidemic in Jamaica, the criminalization of same‐sex sexual relations and a culture of pervasive homophobia have undermined the national HIV response [26, 27]. Previous studies have found that Jamaica's Offenses Against Person Act, which was enacted in 1864, and which the Jamaican Supreme Court declined to overturn in 2023, and widespread police harassment of MSM and transgender women, have resulted in barriers to accessing HIV prevention and treatment services [28, 29]. A 2016 report commissioned by J‐FLAG found that more than half (51.3%) of LGBTQI individuals surveyed who had experienced physical or sexual assault did not report it to the police. Forty‐one percent did not report it because they did not believe the police would take any action [30].

In response to these challenges, the Enabling Environment and Human Rights (EEHR) unit of the Jamaican Ministry of Health and Wellness and civil society organizations (CSOs) such as JASL, JFJ, JN+, Eve for Life, J‐FLAG, Equality for All and Transwave have participated in intensive efforts to present hundreds of legal literacy and “Know Your Rights” sessions for community members throughout the country. Results from the campaign show that people living with HIV have high levels of understanding of their rights and ways to seek redress in the face of discrimination in healthcare, employment and other sectors [25]. In addition, the EEHR unit and CSOs trained more than 1000 police officers in regions across Jamaica on the human rights of people living with HIV and KPs, including pre‐service recruits at the national police academy and in‐service trainings that reached regional and divisional leaders in addition to rank and file officers. Post‐training surveys indicated changed attitudes among police and KPs reported improved treatment [25].

Nonetheless, the organizations leading these programmes noted a gap in national coverage and challenges faced by new organizations seeking to expand knowledge of human rights and HIV. In response, and with the goal to ensure that lessons learned from more established organizations were passed from one generation of activists to the next, J‐FLAG created a series of workshops designed to build visibility and capacity for LGBTQI activists in several regions in Jamaica to fight against stigma and discrimination, complementing training of police with building confidence among LGBTQI communities.

One aspect of the training focused on teaching the history of HIV and the human rights movement. The initiative was designed around a theory of change that learning about the history of the human rights movement in Jamaica could strengthen the sense of solidarity and build collaboration among groups working on HIV despite diversity in terms of generation and geography. The director of a newly founded organization named Queertego, based in Montego Bay, attended one workshop and described the experience of being in a safe, open space where queer people could freely express themselves as profoundly changing his perception of himself and his place in the world [25].

Building on the relationship established in the workshop, and with the backing of a partnership with J‐FLAG and Jamaica AIDS Services for Life (JASL), Queertego was able to significantly expand their community presence by hosting LGBTQI health fairs, town hall events and workshops focused on issues central to LGBTQI people in Montego Bay, including HIV prevention, access to PrEP, substance use and mental health challenges.

The *Breaking Down Barriers* assessment found that Queertego's partnership with J‐FLAG and JASL had improved access to HIV education, prevention and care in Montego Bay while also opening an important connection with JASL's extensive legal network and resources, which includes peer HIV advocates trained as legal “focal points” as well as paralegals and lawyers offering services to people living with HIV in three regions of the country. Expanding the network geographically also strengthened the Jamaican Anti‐Discrimination System and Shared Incidents Database (JADS), a platform for reporting and resolving complaints related to discrimination and human rights violations used by civil society and Jamaican government agencies, including the Ministry of Health and Wellness and key social service and child welfare agencies. More complete coverage and stronger collaboration promoted faster resolution of issues related to HIV prevention and treatment access and offered an opportunity for greater understanding of linkages between human rights programming and health and HIV outcomes [31].

## 3 CONCLUSIONS

Addressing human rights challenges that reduce access to prevention and treatment are critically important to the implementation of successful people‐centred HIV prevention approaches. Integrating programmes targeting stigma and discrimination, improving laws and ensuring rights‐based law enforcement practices, expanding legal literacy and access to justice, reducing gender discrimination and harmful gender norms and mobilizing communities to demand respect for human rights for LGBTQI communities, women, sex workers and others acutely impacted by HIV helps to close the gap to achieve global goals to “end AIDS” by 2030. Especially as human rights environments for KPs deteriorate in many countries, targeted rights‐based programmes to reduce barriers to HIV services and mitigate harm from punitive laws and policies become increasingly urgent.

However, human rights‐related interventions should not be expected to reduce barriers to HIV prevention and treatment overnight, especially in a moment where funds for global health research and implementation, generally and for marginalized populations in particular, have been drastically cut. In March 2025, USAID's staff was cut from about 10,000 employees to 15, before being closed completely on 1 July 2025 [32]. In the next 5 years, cuts to foreign assistance could cause an additional 4–10 million new HIV acquisitions and 1–3 million HIV‐related deaths [33]. It is especially at these times when rights‐based programmes that combat discrimination and criminalization – which cost governments little – are essential to sustaining HIV prevention and treatment gains. While historically rights‐based approaches were supported primarily by external donors, the Breaking Down Barriers initiative required countries to commit matching funds and integrate rights into national strategic plans, fostering sustainability.

A growing body of evidence across a range of diverse countries and settings demonstrates the potential of a model of people‐centred services that recognizes the impact of family, community and the social and political environment on access to HIV services and aims to strengthen advocacy and respect for human rights. Community‐led initiatives can increase access to prevention and treatment services by reducing structural and legal barriers for people disproportionately affected by HIV, particularly criminalized and marginalized populations.

Nearly all of the rights‐based interventions supported by the Global Fund's BDB initiative, as identified in the Box above, address traditional core responsibilities of government: enforcing non‐discrimination; ensuring law enforcement is just and fair; educating the public on rights and ensuring access to justice for all. Recognizing this, government officials have increasingly spoken out about the importance of rights‐based approaches, especially in countries where the HIV response has been most successful.

For example, in Botswana on World AIDS Day 2024, the country's Vice President emphasized the importance of human rights interventions, saying that “…collaboration between stakeholders such as lawmakers, the judiciary, the police, traditional leaders and private legal practitioners must be strengthened with a view to removing human rights barriers” [34]. The Vice President went on to note that the government had established a human rights unit within the National AIDS and Health Promotion Agency (NAHPA).

In 2020, Jamaica became one of the first countries to join the Global Partnership for Action to Eliminate all Forms of HIV‐Related Stigma and Discrimination. The initiative brings together government, civil society, donors and others to address rights‐related issues across sectors, including the workplace, justice system, families and communities. In launching the initiative's first report in June 2021, the Minister for Health and Wellness, Juliet Cuthbert Flynn, reiterated the government's commitment to addressing the social and legal issues that are barriers to an effective HIV response and called for political leadership across party lines to recognize their role in helping to create an enabling environment [35].

Similarly, South Africa's 2023–2028 National Strategic Plan for HIV, TB and STIs mentions “human rights” throughout [36]. Protecting and promoting human rights and advancing access to justice is specifically identified as a key objective, and “reducing inequalities through human rights‐based, people‐ and community‐centred approaches” and removing “all societal and legal barriers” are identified as essential to achieving global HIV targets.

Of course, what is written in plans and said on World AIDS Day is not always what is delivered, and the politics of criminalizing KPs can be more appealing to governments than implementing effective HIV programmes. However, since 2017, the Global Fund's Breaking Down Barriers project has invested more than US$200 million in rights‐based programmes involving both non‐governmental organizations and government agencies in the response, and countries have made matching contributions from their own treasuries [13].

In each of the case studies above, impact and sustainability were fostered by partnerships between community‐based organizations, Ministries of Health and legal professionals. Organizations often prioritized mediation strategies and working outside of formal judicial systems to address and overcome barriers to HIV services, while maintaining the option of seeking accountability through the courts or direct appeals to the government as needed.

In order to achieve AIDS 2030 goals, rights‐based interventions such as those that promote community mobilization, access to justice, and that combat stigma and discrimination are essential, both for KPs and to ensure that people‐centred services are equitable and available to everyone. Donors and governments should recognize their value and expand support for evidence‐based approaches that place people and human rights at the centre of person‐centred HIV prevention and treatment.

## COMPETING INTERESTS

The authors report no competing interests.

## AUTHORS’ CONTRIBUTIONS

MM and JJA conceptualized the article. MM wrote the first draft, and MM and JJA contributed to subsequent revisions.

## FUNDING

This article was written without specific donor funding.

## Data Availability

Data sharing is not applicable to this article as no datasets were generated or analysed during the current study.
